# Attitudes of national decision-makers towards differentiated integration in the European Union

**DOI:** 10.1057/s41295-022-00303-7

**Published:** 2022-05-16

**Authors:** Stefan Telle, Claudia Badulescu, Daniel Fernandes

**Affiliations:** 1 European University Institute, De Wilmskamp 185, 7552GX Hengelo, The Netherlands; 2 European University Institute, Via Fra Jacopo Passavanti, 42, c/o Velucchi, 50133 Firenze, Italy; 3 European University Institute, Via della Badia dei Roccettini, 9, 50014 Fiesole, Italy

**Keywords:** European Union, Differentiated integration, Opt-out, Dependence, Capacity, Identity

## Abstract

Differentiated integration (DI) in the European Union (EU) has mainly been understood as variation in participation in common policies. But DI also has implications for the nature and functioning of the EU as a polity. While temporary DI may facilitate deeper integration, permanent DI is liable to increase transaction costs and fragmentation. However, little is known about how such alternatives are assessed by decision-makers in the member states. This article uses novel quantitative and qualitative data to shed light on this question. It looks at the explanatory role of various types of opt-outs and at member states’ dependence, capacity, and identity. We find that temporary and permanent differentiation are assessed differently in the member states but neither alternative is clearly preferred. Long-term involuntary opt-outs are related to negative assessments of both forms of DI. Surprisingly, voluntary opt-outs do not seem to lead to more positive assessments of DI. We also find that the temporary DI is preferred in smaller member states, while support for permanent DI is higher in larger member states. Finally, we find differences in the effects of dependency, capacity, and identity between older and newer member states.

## Introduction

Differentiated integration (DI) describes a situation in which the ‘territorial extension of European Union (EU) membership and EU rule validity are incongruent’ (Holzinger and Schimmelfennig 2012: 292). In other words, DI means that not all member states participate in all EU *policies* to the same extent and at the same time. Accordingly, differences in the *policy*-specific integration preferences and capacities of the member states are at the core of positive theories on the origins and empirical patterns of DI (Leuffen et al. 2013; Schimmelfennig and Winzen 2020).

However, differentiation also has accumulative effects on the nature and functioning of the EU as a *polity*. This perspective is captured in the concept of the EU as a system of differentiated integration (Leuffen et al. 2013). Depending on the predominant patterns of differentiation, it has been suggested that the system could variously take the form of a ‘Multi-Speed Europe’, a ‘core Europe’, or a ‘Europe à la carte’ (Schimmelfennig and Winzen 2020). While the ‘Multi-Speed Europe’ model suggests that differentiation is a temporary phenomenon, the ‘core Europe’ and ‘Europe a la carte’ models imply the possibility of permanent polity differentiation. While transaction costs and the risk of fragmentation may be lower when DI is temporary, permanent DI is arguably better suited for accommodating deep seated sovereignty concerns.

For these reasons, we would expect that the models differ in their appeal to decision-makers in the member states. However, little is known about how national decision-makers assess these different varieties of what we call *polity differentiation*. In this paper, we ask two questions: First, what attitudes do decision-makers in EU member states have towards temporary and permanent differentiation? Second, what explains their attitudes? We use novel quantitative and qualitative data gathered in the framework of the InDivEU project to answer our questions.

Despite the generally low salience of DI in national political debates, we find variation in how DI is seen in the member states. Overall, both temporary and permanent DI are seen slightly negatively. In member states with enduring involuntary opt-outs, both types of differentiation are seen more negatively than in other member states. Surprisingly, however, voluntary opt-outs do not necessarily lead to a more positive assessments as policy-specific preferences for non-integration often conflict with a polity preference for maintaining influence in the EU. Temporary differentiation is seen more positively in smaller member states than in larger member states. Support for permanent differentiation seems to be higher in older member states which are larger and have a high share of citizens identifying as exclusively national.

The paper is structured as follows. The next section builds on the concept of the EU as a system of differentiated integration and explains what we mean by polity differentiation. We then review the existing literature and formulate expectations regarding national decision-makers attitudes towards polity DI. The second section presents our data and methods of analysis. The third section presents the empirical findings. The fourth section summarizes our findings and discusses implications, limitations, and potential further research trajectories.

## Theoretical framework: systems of differentiated integration

The aim of this article is to understand what political decision-makers in EU member states think about differentiated integration. For the purpose of this article, we view members of a national government as well as parliamentarians as political decision-makers. We build on work by Leuffen et al. (2013) who conceptualize the EU as a *system of differentiated integration* which is defined as ‘one Europe with a single organizational and member state core and a territorial extension that varies by function’ (Schimmelfennig et al. 2015: 767).

In this perspective, differentiation of the EU polity can occur along various dimensions. Leuffen et al. (2013) distinguish between policy centralization (differences in the intensity of cooperation between policies) and policy extension (differences in the participation of member states in EU policies). Similarly, Holzinger and Schimmelfennig (2012) distinguish between functional and territorial differentiation. In addition, they identify five further analytically distinct dimensions (Table 1). Differentiation can either be temporary or permanent (also see Stubb 1996). It can occur exclusively between EU member states (internal differentiation) or also involve non-member states (external differentiation) (also see Gstöhl 2015; Lavenex 2015). It can be restricted to differences at the member state level, or also involve differences between sub-units of member states. It may be treaty-based or take the shape of intergovernmental agreements (also see de Witte 2018, 2019). Finally, a differentiated EU may retain EU-wide decision-making processes or decision-making may happen in a decentralized fashion within intergovernmental clubs.

**Table 1:** Dimensions of polity DI

Dimension	Distinction
Policy territorial extension	More vs. less inclusive policy membership
Policy centralization	More vs. less centralized policy
Time	Temporary vs. Permanent
EU exclusivity	Only EU members (internal DI) vs. also non-members (external DI)
Levels	Nation-level differentiation vs. multi-level differentiation
Legal basis	Only inside EU Treaties vs. also outside EU Treaties
Decision procedure	EU decision-making vs. club decision-making

Own compilation. Based on Leuffen et al. (2013) and Holzinger and Schimmelfennig (2012)

Building on the first three dimension in Table 1, Schimmelfennig and Winzen (2020) distinguish three alternative models of differentiated integration in the EU. In the ‘*Multi-Speed Europe’* model differentiation is a temporary phenomenon as exceptions from common rules get phased-out over time. The ‘*Multi-tier Europe*’ model describes the existence of a gap between a more integrated core group of member states and a less integrated periphery. Finally, the ‘*Multi-menu Europe’* model describes the existences of overlapping policy regimes with varying membership, as member states can freely ‘pick and choose’ policy fields they wish to participate in.

For our analysis, we conflate the ‘Multi-tier’ and ‘Multi-menu’ models into what we call ‘Multi-end Europe’. There are conceptual and practical reasons for this. Conceptually, the main difference between the ‘Multi-tier’ and the ‘Multi-menu’ models is the presence or absence of an organizational and membership core.^1^ However, what they have in common is more important: both models question the fundamental idea of an ever-closer union and imply, instead, that European integration can lead towards multiple different endpoints. By contrast, the ‘Multi-Speed Europe’ model is compatible with the prospect of an ever-closer union. In this sense, therefore, the difference between temporary and permanent differentiation is a fundamental one. The second reason is practical. As the three models are scholarly constructs, it appears unlikely that national politicians would draw clear distinctions between all three of them. However, we expect that they may distinguish between temporary and permanent differentiation because it is easy to grasp and has clear and important implications. In the following two subsections, we develop several theoretical expectations regarding how decision-makers think about DI.

- *Normative desirability and the role of opt-outs*

In a first step, we look at how the different characteristics of ‘Multi-Speed’ and ‘Multi-End’ differentiation may influence their assessment by national decision-makers. Bellamy and Kröger (2019) have discussed the conditions under which differentiated integration could be seen as a fair scheme of cooperation between democratic states. The key criteria are the efficient provision of transnational policies and the safeguarding of member states’ and citizens’ capacity for self-determination. In other words, differentiated integration is normatively desirable to the extent that it positively impacts the output and input legitimacy of the EU. In terms of output legitimacy, we can distinguish between the effect of DI on decision-making efficiency, on transaction costs in the EU, and on the likelihood of further fragmentation or disintegration. Regarding input legitimacy, we can distinguish between voluntary and involuntary differentiation (Table 2).

**Table 2:** Output and input legitimacy of ‘Multi-Speed Europe’ and ‘Multi-end Europe’

Criteria	Sub-criteria	Multi-Speed Europe	Multi-End Europe
EU functioning (Output legitimacy)	Decision-making	Higher efficiency	Higher efficiency
	Transaction costs	Transient increase	Accumulative increase
	Disintegration	Low risk	Higher risk
Self-determination (Input legitimacy)	Voluntary DI	High	High
	Involuntary DI	Low	Very low

Own compilation. Based on Bellamy and Kröger (2019); Schimmelfennig and Winzen (2014); Kölliker (2001); Fossum (2015)

We begin by looking at how the two models affect the functioning of the EU. In terms of decision-making efficiency, both models do equally well. Differentiation contributes to more efficient decision-making by increasing the homogeneity of preferences among the decision-makers. In this context, it does not matter whether the group of decision-makers is in principle open to others or not. In terms of transaction costs, however, ‘Multi-Speed Europe’ performs better because increases of transaction costs due to differentiation are only temporary. By contrast, the ‘Multi-end Europe’ model implies permanently increased transaction costs. Permanent status differences among member states also lend themselves to Eurosceptic political mobilization as an electoral strategy (i.e. ‘Second-class membership’) and thus increase the risk of disintegration. Overall, increased transaction costs and a higher risk of disintegration result in a lower output legitimacy of the ‘Multi-end Europe’ model.

**H1**: Attitudes towards the ‘Multi-Speed Europe’ model are more positive than towards the ‘Multi-End Europe’ model.

Regarding the input legitimacy of the two models, the crucial question is whether differentiation is voluntary or involuntary. Voluntary differentiation improves self-determination because it allows member states to selectively opt-out of those European policies for which there is no domestic support. As long as this is a voluntary choice of the member state, it does not matter for the input legitimacy of the EU whether differentiation is temporary or permanent.

Involuntary differentiation is harder to square with self-determination. This is especially the case when involuntary differentiation is permanent or involves negative externalities for the involuntary outgroup (Kölliker 2001). To the extent that the involuntarily excluded member state is negatively affected by policies over which it had no say, differentiation can lead to domination (Bellamy 2011; Fossum 2015). Involuntary differentiation typically occurs in the context of EU enlargements to ‘exclude the new member states temporarily from desired rights and benefits of EU membership’ (Schimmelfennig 2014: 682). It can be assumed that new member states view temporary exclusions as the price to pay for an earlier EU accession. However, should these exclusions become perceived as permanent, we would expect decision-makers in the affected member states to become highly sceptical of any kind of differentiation. In this scenario, it seems plausible that involuntary differentiation will no longer be seen as an ‘express ticket’ towards full EU membership, but as a ‘slippery slope’ to permanent ‘second-class’ membership. Involuntary differentiation can become quasi-permanent when the full participation of a member state in an EU policy gets blocked by the veto of another member state in the European Council. The prime example is the continued exclusion of Romania, Bulgaria, and Croatia from the Schengen Area, despite positive technical assessments of their capacity to join (European Parliament 2011). In sum, we expect that the type of non-participation impacts how national decision-makers assess the two models:

**H2a**: Attitudes towards the ‘Multi-End Europe’ model are more positive in member states with voluntary opt-outs than in member states without voluntary opt-outs.

**H2b**: Attitudes towards the ‘Multi-Speed Europe’ and the ‘Multi-end Europe’ models are more negative in member states which are subject to involuntary opt-outs than in member states without involuntary opt-outs.

**H2c**: Attitudes towards the ‘Multi-Speed Europe’ model are more positive in member states which are no longer subject to involuntary opt-outs than in member states which never were subject to involuntary opt-outs.

(b) *Member state dependence, capacity, and identity*

Schimmelfennig and Winzen (2020) suggest that differences in policy integration preferences of national governments are rooted in the heterogeneity of member states regarding their dependence, capacity, and identity. The intergovernmental character of their theoretical framework implies that integration preferences are *policy*-specific. We are, however, interested in attitudes towards different varieties of *polity* differentiation. Hence, we use their framework as a starting point for developing expectations regarding the EU as a differentiated polity.

Beginning with dependence, Schimmelfennig and Winzen build on realist intergovernmentalism to suggest that larger member states are less dependent on achieving integration outcomes because they can ‘go it alone’ more often than small member states. This view contrasts with the argument that EU decision-making gives less voice to smaller member states (i.e. QMV), which may, therefore, view DI as a way of maintaining autonomy (Eriksen 2018, 2019). It has, however, also been argued that the community method and a unanimity bias in the Council protect and even amplify the voice of smaller member states(Scharpf 2016). As such, we would expect that:

**H3**: Attitudes towards the ‘Multi-Speed Europe’ and the ‘Multi-end Europe’ models are more negative in smaller member states than in larger member states.

**H3b**: In smaller member states, the ‘Multi-End Europe’ model is seen even more negatively than the ‘Multi-Speed Europe’ model.

A similar logic applies to differences in the capacity of member states. The basic assumption is that richer states prefer higher regulatory standards and poorer states prefer lower standards. This suggests that poorer member states have an incentive to seek voluntary exemptions from demanding EU policies (i.e. environmental standards), but may also be subject to involuntary exclusions from EU policies (i.e. free movement). In fact, research by Frank Schimmelfennig (2014) suggests that voluntary exemptions are historically more prevalent than involuntary exclusions. Nevertheless, is appears plausible to assume that political decision-makers in poorer member states will view DI overall rather negatively because ‘full EU membership’ is an objective in its own right, promising international prestige and domestic modernization. This suggests that:

**H4**: Attitudes towards the ‘Multi-Speed Europe’ and the ‘Multi-end Europe’ models are more negative in poorer member states than in wealthier member states.

**H4b**: In poorer member states, the ‘Multi-End Europe’ model is seen even more negatively than the ‘Multi-Speed Europe’ model.

Regarding identity, Schimmelfennig and Winzen build on post-functionalism to suggest that member state demand for integration is dependent on public opinion.^2^ Post-functionalism assumes that national decision-makers are responsive to—and constrained by—public opinion about European integration. Regarding differentiated integration, this leads to the assumption that member states with a greater share of citizens identifying exclusively with their nation will be less integration-seeking and view DI as a way to protect national sovereignty (Winzen 2016). As identities tend to be stable over time, such member states should find the possibility of permanently opting out of EU policies particularly attractive. By contrast, high shares of pro-European citizens would make a government more likely to perceive the ‘Multi-Speed Europe’ model as a way to move integration forward without jeopardizing the idea of an ever-closer union.

**H5**: Attitudes towards the ‘Multi-End Europe’ model are more positive in member states with high shares of citizens identifying as exclusively national than in member states with higher shares of citizens also identifying with the EU.

**H5b**: Attitudes towards the ‘Multi-Speed Europe’ model are more positive in member states with high shares of citizens identifying also as European than in member states with higher shares of citizens identifying as exclusively national.

## Data and methods

To our knowledge, there are no systematic data on how national decision-makers think about different models of polity differentiation as most existing DI research has focused on the policy integration preferences of the member states (but see Moeller et al. 2017). To address this gap, we collected new qualitative and quantitative data by analysing key governmental and parliamentary documents. In comparison to elite interviews or surveys, the advantage of document analysis is that it is based on actual positions which were expressed by members of governing parties without being prompted by the researcher. Moreover, the frequency of references to differentiated integration in governmental documents also serves as a measure of the salience of DI.

Data collection was carried out in several stages. First, we established a list of keywords^3^ (Appendix 1) relating to the two models of polity differentiation and we identified the categories of key documents to be analysed: i.e. government programmes, key speeches by heads of state and government in the national and European political arena, and Parliamentary debates (see Appendix 2 for a detailed overview of the documents analysed). Parliamentary minutes were included in the analysis for two reasons: first, parliamentary minutes enable a more fine-grained mapping of political opinions below the level of official government positions. Second, as we expected low number of references to DI in official government documents (which proved correct), the richness of parliamentary minutes would allow the researchers to base their evaluations of governmental positions on more data points.

We then recruited researchers for all 27 EU member states and tasked them with translating the keywords into the relevant local languages and search for them in the respective national documents. Only parliamentary debates yielded a significant number of search hits. All references by member of governing parties were coded by the country researchers as either positive, neutral, or negative. To ensure that they did not miss indirect references to DI, the researchers were asked to closely read governmental programmes and speeches. This confirmed that DI was usually not a salient issue in these documents.

The country researchers then produced detailed qualitative country reports based on their findings from the document analysis. These reports serve as the source of the qualitative data in this paper. In addition, to facilitate comparisons between countries, we also asked the country researchers to complete a survey in which we asked how governments assessed the ‘Multi-Speed Europe’ and ‘Multi-end Europe’ models. The survey used a 5-point scale as proposed by Leruth (2015) to study EU integration preferences of parties or governments. The scale runs from (1) very negative, (2) negative, (3) neutral, (4) positive, to (5) very positive. We cross-checked the answers we received from the researchers with an internal coding of the positions which was based on the 27 country reports. Remaining disagreements were resolved through deliberation between the authors of the country reports and the internal coder. In this way, we obtained one score per member state and DI model for the period 2008–2019. Due to the low number of DI-related references, it was decided to aggregate all references in the period 2004–2019 into one overall score. While this approach conflates the positions of changing governments within one country, it nevertheless provides a more accurate picture of how DI is seen *on* average within EU member states. In addition, it is plausible to assume that governing parties within a country do not strongly vary in their assessment of DI due to the generally low salience of European integration to voters and because structural factors (i.e. country size, wealth, identity) condition governments to adopt similar positions.

Turning to the independent variables, we first discuss how we assigned member states to different categories of opt-outs. We did so based on whether a member state is/was voluntarily exempted or involuntarily excluded from the adoption of the common currency and from participation in the Schengen Area. A focus on these two policies is justified for two reasons. First, exceptions from these two policies are seen as ‘the most substantively significant for the future of European integration’ (Jensen & Slapin 2012: 786). Second, together with the internal market policies, they account for more than 90 per cent of differentiations (Duttle et al. 2017: 409, see also 420). We furthermore assume that the most likely cases to show an effect are those countries which have/had (in)voluntary opt-outs in *both* policies. Denmark is the only EU member with voluntary opt-outs from Schengen and the Eurozone. Therefore, we also include Ireland (Schengen opt-out) and Sweden, Czechia, Hungary, and Poland (de facto opt-out from the Eurozone). Table 3 summarizes the selected cases (see Appendix 3 for more details).

**Table 3:** Case selection for effect of different opt-out types

Expected Effect	Multi-Speed Europe (MSE)	Multi-End Europe (MEE)
Positive MSE—MS with ceased involuntary opt-outs MEE—MS with voluntary opt-out	**Malta** **Slovakia** **Slovenia** **Latvia** **Lithuania** **Estonia** **Greece**	**Denmark** Ireland *Sweden* *Czechia* *Hungary* *Poland*
Negative MS with ongoing involuntary opt-out	**Romania** **Bulgaria** **Croatia**	**Romania** **Bulgaria** **Croatia**

Own compilation. Bold = opt-out from both Schengen and Eurozone; Italics = de facto opt-out from Eurozone

For the variables of size, wealth, and identity, we followed Schimmelfennig and Winzen (2020). We operationalized size as the size of the economy as measured in overall GDP. This variable was logged, given that absolute differences in size between two small countries should be more impactful than those same differences between large countries.^4^ For wealth, we used GDP per capita.^5^ For identity, we used Eurobarometer data^6^ on the share of the population identifying as ‘[Nationality] only’. In each case, we used the average value for the period 2010–2019.

We use our survey data to plot relationship between the assessment of the two models and our independent variables (opt-out status, size, wealth, and identity). Due to the low number of data points, we then complement the survey data with our rich qualitative data from the 27 country reports. In other words, we use ordinal differences in our variables of interest to pre-structure a narrative country comparison. According to James Mahoney (1999), the combination of ordinal and narrative methods allows researchers to consider the interactions between multiple contextual factors. The advantage is a less deterministic view of the interaction between the variables of interest. The disadvantage is a loss of parsimony.

## Results

In this section, we present our findings. In “Dependence: small states prefer temporary and large old states prefer permanent differentiation” section, we show how the two models are assessed by political decision-makers in the member states. In section “Capacity: capacity matters in the new member states”, we discuss the effect of different types of opt-outs. In section “Integration preference: identity matters in the old member states”, we discuss the effect of member state size, wealth, and identity. However, before doing so, a short disclaimer on the salience of DI is needed to contextualize our results. The documents analysis revealed that DI was not a very salient issue in political debates in the member states in the period of investigation. While we found references to DI in parliamentary minutes (see Appendix 4), they were largely absent from government programmes or key speeches by heads of state and government. The main takeaway from these findings is that we need to be cautious in assuming that member state governments have clearly specified and stable positions about polity differentiation. For this reason, the following sections always contextualize the aggregate assessment of the two models of polity DI.

### Attitudes towards ‘Multi-Speed Europe’ and ‘Multi-End Europe’

Table 4 presents an overview of governmental attitudes towards ‘Multi-Speed Europe’ and ‘Multi-End Europe’. Of the 26 member states^7^ for which we obtained a score on the ‘Multi-Speed Europe’ model, only four member states have a favourable view, ten have a neutral position, and twelve countries view it negatively or very negatively. By comparison, six member states view the ‘Multi-End Europe’ model positively, five have a neutral position, and thirteen countries view it negatively or very negatively.^8^

**Table 4:** Attitudes towards ‘Multi-speed Europe’ and ‘Multi-end Europe’

Country	Multi-Speed	Multi-End
Austria	Negative	Negative
Belgium	Neutral	Negative
Bulgaria	Very negative	Very negative
Croatia	Positive	n/a
Cyprus	Neutral	Negative
Czechia	Neutral	Neutral
Estonia	Neutral	Neutral
Finland	Positive	Neutral
France	Negative	Positive
Germany	Neutral	Very negative
Greece	Neutral	Neutral
Hungary	Neutral	Negative
Ireland	Negative	Negative
Italy	Negative	Positive
Latvia	Positive	Neutral
Lithuania	Negative	Negative
Luxembourg	Positive	Very negative
Malta	Neutral	Positive
The Netherlands	Neutral	Positive
Poland	Negative	n/a
Portugal	Negative	Negative
Romania	Very negative	Very negative
Slovakia	Neutral	Positive
Slovenia	Negative	Negative
Spain	Negative	Negative
Sweden	Negative	Positive

Based on survey among authors of country reports

Even though, on average, the multi-speed model is viewed slightly more positively than the multi-end model,^9^ the difference in the assessments of the two models is small. Hence, the data do not support hypothesis 1. Nevertheless, the analysis shows that politicians draw a distinction between the two models in a majority of member states. More important than the difference in the assessment of the two models is the fact that both models were assessed in a slightly negative fashion on average and that only a small group of countries has positive views about either model. Moreover, no member state assesses both models positively, but several member states assess both models (very) negatively. This suggests that, overall, polity differentiation is ‘second best’ to uniform integration. What explains these differences?

### Voluntary and involuntary opt-outs

**Fig. 1:**
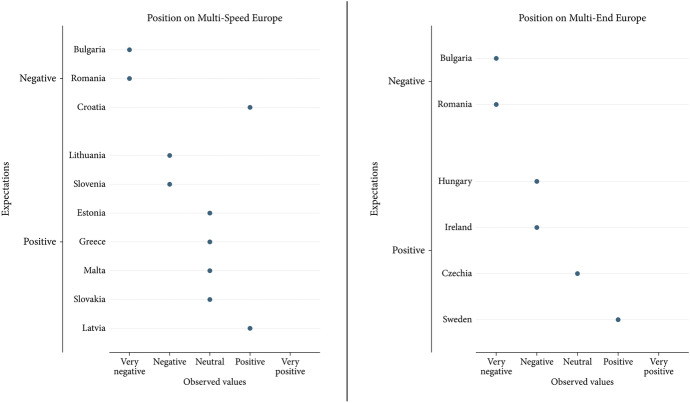
Opt-out types and assessments of polity DI

Figure 1 gives an overview of how well our expectations regarding the role of voluntary and involuntary opt-outs were met. It lends support to the expectation that long-term ongoing involuntary opt-outs do affect assessments of polity differentiation negatively. However, there is less evidence for the role of past temporary opt-outs and of voluntary opt-outs. In the following, we use our qualitative data to tease out why this might be the case.

#### Voluntary opt-outs: balancing the costs and benefits

We find little evidence for the expectation (H2a) that governments of member states with voluntary opt-outs assess the ‘Multi-End Europe’ model more positively. Using our qualitative data, we zoom in on the countries in this group. In all cases, this analysis revealed a friction between the choice to opt-out of an EU *policy* and the expected negative *institutional consequences* of that choice in terms of losing influence in the EU. Importantly, the positive and negative consequences of a voluntary opt-out appear to often cancel each other out, leaving no clear effect on the assessment of ‘Multi-End Europe’ model.

Beginning with Denmark, our qualitative data suggest that before the 2015 referendum on ending the Danish opt-out in home and justice matters, Danish governments were actually ‘working to abolish or ease up the Danish opt-outs’ (Sand Madsen 2020: 18).^10^ Indeed, only the more Eurosceptic Social Democratic government of Mette Frederikson (since 2019) ‘did not position itself clearly negatively towards opt-outs […] indicating a policy shift compared to previous periods’ (Sand Madsen 2020: 18). Hence, previously more integrationist elite attitudes appear to have aligned with more integration-sceptic popular attitudes in Denmark.

Looking at Ireland, our qualitative data reveal that the Irish opt-out from Schengen was driven by the pragmatic decision to maintain the Common Travel Area with the UK after that country’s opt-out of Schengen. In addition, the negative outcome of the first Irish Lisbon Referendum created intensive fears that the other member states might go ahead without Ireland, creating a ‘two-tier Europe’. This fear was vividly expressed by Deputy Niall Collins of the governing Fianna Fail Party on 18 June 2008, one week after the referendum:

‘Worryingly, there is an emerging scenario against which we must guard at all costs, namely some form of two-speed or two-tier system developing, irrespective of whichever option is decided on ultimately. It would be an unmitigated disaster were Ireland to end up with second-class membership of Europe in a slower lane. Undoubtedly, it would be with reduced influence and goodwill and without the political firepower to defend our vital national interests’ (Niall Collins, Parliament, 18 June 2008; cited in Telle 2020: 14).

Thus, the negative Irish assessment of any differentiation derives from an expected loss of influence in Europe resulting from the country’s pragmatic alignment with the UK and popular opposition to deeper integration. Tellingly, after the UK’s withdrawal from the EU, Ireland has joined both Pesco (in 2017) and the Schengen Information System (in 2021).

Next, we turn to those member states which have de facto voluntary opt-outs from the Eurozone. Sweden is the only country where the ‘Multi-End Europe’ model was seen positively. But even here, this positive view needs to be qualified as the positive assessment is largely based on ‘the Swedish ambition to be in the core of Europe rather than the general concept of a core Europe’ (Herolf 2021: 14). In fact, this ambition is rooted in worries about diminished Swedish influence in Europe in the context of a widening gap between and increasingly integrated Monetary Union and the non-Euro states.

Similarly, in the Czech Republic, the ‘Multi-End Europe’ model is assessed neutrally, signifying how ‘Czechia is learning with difficulty how to square two elements: being outside the eurozone and yet not being outside the core of Europe’ (Hlavik and Smekal 2020: 16). In Poland, despite the fact that Eurozone membership has not been a political priority, governments perceived a ‘need to enter the core of Europe to overcome the tendency of a narrow circle of EU countries to make decisions’ (Walecka and Gagatek 2021: 12). Finally, in Hungary, the Fidesz government assessed the ‘Multi-End Europe’ model negatively because it is associated with ‘Second-class membership’, while the ‘Multi-Speed Europe’ model is seen in terms of desirable flexibility. This position is illustrated by a statement the Deputy State Secretary Balázs Péter Molnár (Fidesz) made in response to the European Commission’s White Paper on the Future of Europe in the European Affairs Committee in on 16 October 2017:

‘I think that, unfortunately, we have to talk about core Europe and the periphery, because there is a realistic chance that a core Europe will emerge. […] if implemented as set out in the Macron plans, it could also result in a parallel institutional structure. […] We believe that Option 3 [of the Commission White Paper], which means a multi-speed Europe with a focus on enhanced cooperation, would provide the flexibility for the current development of the European Union that the Member States could take advantage of’ (Balázs Péter Molnár, European Affairs Committee, 16 October 2017; cited in Kyriazi 2021: 14–15).

Overall, the above discussion revealed that countries with voluntary opt-outs from major EU policies are surprisingly torn over their decision, indicating a difficult trade-off between desired *policy* differentiation and undesired *polity* differentiation.

#### Ongoing involuntary opt-outs: technical readiness vs. political dynamics

Romania, Bulgaria, and Croatia are subject to ongoing involuntary opt-outs from both the Schengen Area and the Eurozone. In these countries, both the ‘Multi-End Europe’ and the ‘Multi-Speed Europe’ model much more negatively than on average among all member states, thus lending support to hypothesis H2b. Nevertheless, the results for Romania and Bulgaria are clearer than those for Croatia, suggesting that longer durations of involuntary opt-outs lead to more negative attitudes towards differentiation.

Romanian governments and opposition parties have consistently and vehemently rejected all types of differentiation as undesirable. This assessment relates to their equally consistent objective of joining the Schengen Area and the Eurozone. With regard to the Schengen Area, the ‘protracted accession to Schengen has come to be seen by Romanians as a symbol of European differential treatment and distrust in the country’s capacity to protect the EU’s external borders’ (Badulescu 2021: 2). A major reason for this view is the fact that the country was assessed to be technically ready to join the Schengen Area as early as in 2011, and ever since has been ‘caught in an indeterminate state between having fulfilled the technical requirements and becoming a fully-fledged member of the Schengen area’ (Badulescu 2021: 2). In line with this, Romanian President Klaus Iohannis stated at the informal European Council of March 2017 in Rome that:

‘We believe, and we have said this very clearly and firmly, in a strong united supportive European Union, and this consolidation must be done together, by all 27. For this reason, I did not think it was right for us to go for variants such as Europe with several speeds or Europe with two speeds or Europe with concentric circles. [...] This has been and will continue to be presented as Romania's position [...]. What is important is not to close variants that can be started by some and that can be reached by all. It is important not to develop exclusive projects. It would be totally counter-productive if the European Union accepted projects that are accessible only to some members and not to others. In fact, that would mean two-speed Europe, and we don't want that at all. We find that we now have such projects but they must represent intermediate phases, transition phases and must represent exceptions, by no means the rule’ (Klaus Iohannis, Informal European Council, 25 March 2017; cited in Badulescu 2021: 19)

The statement shows that ‘Two-Speed Europe’ is understood as a potentially permanent condition by the Romanian president. It indicates a pervasive suspicion that differentiations which begin as temporary exclusions may become permanent due to the political dynamics in the EU. As a consequence, *both* models are seen very negatively by Romanian decision-makers. In Bulgaria, the situation is very similar. As Markova (2020) states in her report,

‘differentiated integration models such as two-speed/multiple speeds Europe are perceived by Bulgarian politicians as a threat to equal participation or fertile ground for growing discrepancies. In the rhetoric of national politicians, “core Europe” is used in parallel with “periphery”, triggering negative associations with second-class membership in a union of more developed and powerful countries’ (Markova 2020: 14).

Croatia, which joined the EU in 2013, contradicts our expectations. In Croatia, the authors of the country report find that ‘DI models have positive mentions, especially in the eyes of the ruling party, while significant negative connotations characterize the opposition’s perception’ (Beroš and Gnip 2020: 10). Importantly, the Croatian discussion was exclusively focused on the idea of a ‘Multi-Speed Europe’, while concepts which refer to potentially permanent differentiation were completely absent from the political discourse. On closer inspection, it becomes clear that the same worries as in Romania and Bulgaria are present in the Croatian debate on DI. However, as the following statement by Prime Minister Andrej Plenković illustrates, ongoing exclusion of Croatia is seen as rooted in lacking domestic capacity, rather than external political factors:

‘In other words, we have to avoid an amalgam in which a few powerful, wealthy and typically founding states will pursue closer cooperation, while the rest will remain at the margins with a decreased influence on the formation of the European project. In my opinion, this is one of the most important facts for Croatia, that *because of the early stages of the evaluation* of our Schengen membership, and that by signing the accession treaty we have formally agreed to become a eurozone member, and that *we still have many criteria to fulfil* on which we are working—that we are not left via facti within something of a firewall excluded from those that make decisions’ (Andrej Plenković, Parliament, 15 March 2017; cited in Beroš and Gnip 2020: 12, emphasis added).

Hence, the more positive assessment of the ‘Multi-Speed Europe’ model in Croatia might be explained by the fact that less time has passed since accession the EU and that the ongoing involuntary opt-outs from Schengen and the Eurozone are not (yet) seen as politically motived.

#### Ceased involuntary opt-outs: becoming and remaining a ‘core’ member state

We find limited evidence for the expectation (H2c) that governments of member states which successfully overcame involuntary opt-outs assess the ‘Multi-Speed Europe’ model more positively than the average. They also view the ‘Multi-End Europe’ model more positively.

Seven countries were temporarily excluded from both the Schengen Area and the Eurozone, but have since joined both: Latvia, Estonia, Lithuania, Slovenia, Slovakia, Greece, Malta. Latvia is the only country in this group, where the ‘Multi-Speed Europe’ model is assessed positively. As a small country on the Eastern periphery of the EU, Latvian governments were eager to quickly overcome the country’s exclusion from Schengen and the Eurozone and joint the EU’s core. Accordingly, the authors of the Latvian report concluded that.

‘Latvia’s [initially negative] outlook on DI in the EU experienced adjustments when the […] country joined the eurozone in 2014. Since then and in the context of Brexit, in discussions on completion of EMU and debates on the Future of Europe in general, Latvia’s position adjusted to the new reality the country was in and it became more accepting of DI as a possible solution’ (Bukovskis et al. 2021: summary of results).

This development was also observed by the author of the country report on Estonia, where the attitude towards differentiation was ‘clearly the most negative’ immediately after accession and ‘driven by a fear of being left behind in the process of integration against its own will’. However, ‘[d]uring the 2010s this fear dissolved and attitudes became more positive, as Estonia started seeing itself as one of the countries that could move on faster and join the core of Europe’ (Reiljan 2020: 9). This was linked to the country’s accession to the Eurozone in 2011. Moreover, as the country emerged quickly ‘from the economic crisis[,] the concept of core Europe and Estonia being part of it was embraced more’ (Reiljan 2020: ibid.).

In Lithuania, by contrast, the negative assessment of the two models have not improved after the country’s accession to the Schengen Area (2007) and the Eurozone (2015). Instead, the authors of the country report for Lithuania found that.

‘parliamentary debates and positions of key foreign policymakers like the President and the Minister of Foreign Affairs […] indicate a continuity in the negative attitude to ‘two-speed’ or ‘several-speed’ Europe. The negative position was based on the assumption that it could potentially lead to disintegration of the EU or could be used as a tool for core EU Member States to marginalise new smaller members’ (Vinogradnaitė and Vilpišauskas 2020: 20).

Importantly, no clear distinction seems to be drawn between the two models in Lithuania (Vinogradnaitė and Vilpišauskas 2020: 11). Both, the unchanged position and the conflation of temporary and permanent differentiation may be reflections of the very low salience of DI in Lithuanian political debates (see Appendix 3).

The negative assessment of ‘Multi-Speed Europe’ is shared by governments in Slovenia. Here it is driven by the fear that the country could lose its status as a core country. In August 2018, Prime Minister Marjan Šarec stated that.

‘we can observe in praxis a two-tier Europe already. The EU will have to find the answers to the common questions as soon as possible, since without them the future may be bleak. Slovenia needs to struggle towards remaining within the so called “core countries”’ (Marjan Šarec, Inaugural Address in Parliament, 17 August 2018; cited in Udovič and Bučar 2021: 12).

Moreover, in the assessment of Prime Minister Janez Janša, the debate on the Future of Europe has been initiated by the big member states to gain more power by increasing the use of majority voting. Against this trend, PM Janša has suggested that Slovenia would use its Council Presidency in 2021 to ‘defend’ the Lisbon Treaty ‘as a basis for the EU, enabling relative equality of the Member States’ (Janez Janša, Inaugural Address in Parliament, 13 March 2020; cited in Udovič and Bučar 2021: 12).

In Slovakia, there was little debate of differentiated integration prior to 2017. Since then, debates have centred around the question of the country’s continued membership in the EU’s core. As such, in 2017, Prime Minister Robert Fico suggested that it is ‘our vital duty to be in the core’ (Parliament, 15 June 2017; cited in Janková 2021: 18) and State Secretary Ivan Korčok explained that ‘membership of the core rests on membership of the eurozone and Schengen’ (Parliament, 22 March 2017; cited in Janková 2021: 18). One year later, in 2018, Minister of Foreign and European Affairs, Miroslav Lajčák explained that the ‘creation of this core EU is not Slovakia’s preferred choice, as the primary interest is common progress. However, if such a core is created, Slovakia shall be part of it’ (Parliament, 11 April 2018; cited in Janková 2021: 18). Hence, the positive assessment of the ‘Multi-End Europe’ model seems to be related to the perception of Slovakia as a stable ‘core country’.

Finally, the Greek experience of involuntary exclusion from Schengen and the Eurozone lies further back in time and does not seem to play an important role in how Greek governments assess differentiated integration. Instead, assessments are shaped by the perceived dominating treatment of Greece by the ‘core countries’ during the Euro crisis. While the relationship was seen as extremely confrontational under the SYRIZA government (2015–2019), the Nea Dimokratia government of Prime Minister Kyriakos Mitsotakis (since 2019) has sought to re-establish Greece as a member of the ‘core countries’ (Tellidou 2021).

The main takeaway from this group of countries is twofold: First, the existence of a ‘core Europe’ is widely perceived as a fact. Assessments of differentiated integration seem to be explicable by the perceived relative movement of a country towards the core (more positive) or away from the core (more negative). Second, the perceived political power of the ‘core countries’ and ‘big member states’ in influencing EU decisions impacts how these governments perceive DI. In other words, not only actual differentiation, but also perceived power inequalities between the member states seem to influence assessments of polity differentiation.

### Dependence, capacity, and identity

This section looks at whether and how the dependence, capacity, and identity of member states play a role in assessments of polity differentiation. We found that small states are more likely to prefer temporary differentiation, while old large states prefer permanent differentiation (3.3.1). Capacity appears to matter in the new, but less so in the old member states (3.3.2). The opposite is the case regarding identity, which seems to matter in the old member states (3.3.3).

#### Dependence: small states prefer temporary and large old states prefer permanent differentiation

**Fig. 2:**
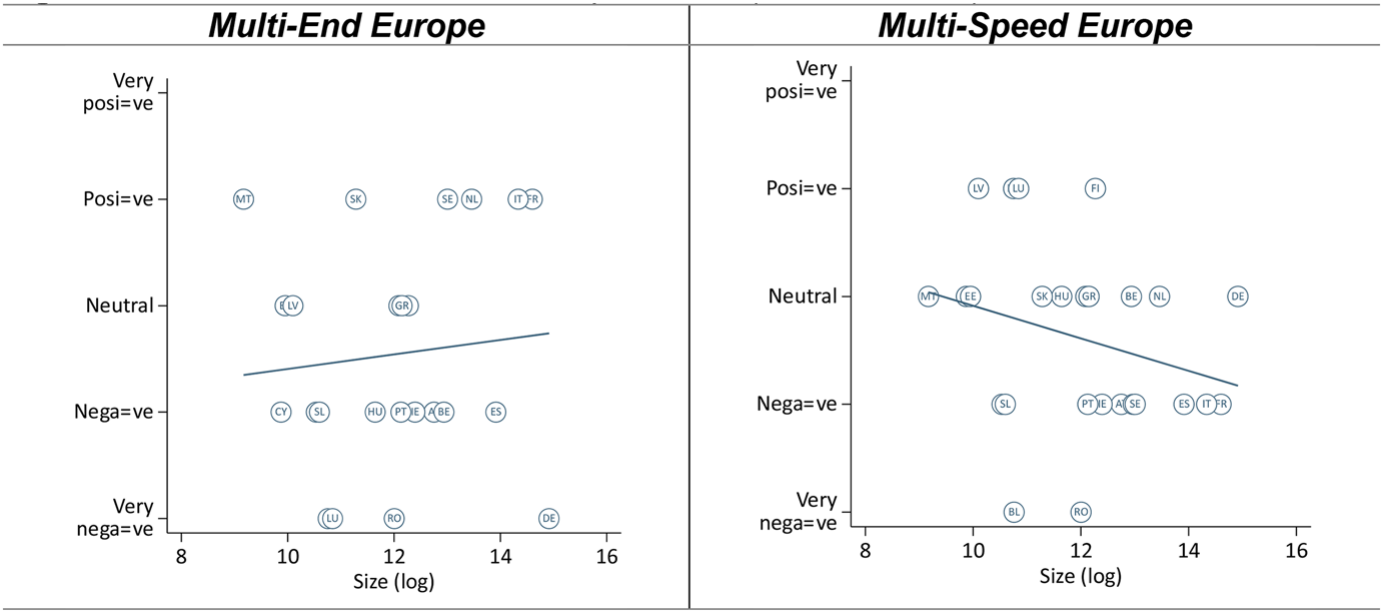
Effect of member state dependence (economic size)

In terms of dependence, we expected that larger member states would assess both models more positively than smaller member states, because the former need integration less (H3a). The findings suggest that larger member states only view the ‘Multi-End Europe’ model more positively, but smaller member states view the ‘Multi-Speed Europe’ model more positively (Fig. 2). Notwithstanding, smaller member states appear more sympathetic with the ‘Multi-Speed Europe’ model over the ‘Multi-End Europe’ model, while the opposite is true for larger member states (H3b, Appendix 5).

The relationships would be stronger for both models if Germany’s negative position was in line with the positive position of other large member states, such as France or Italy. Opt-out experience does not explain the differences between these countries, as neither of these states had any opt-outs from major EU policies. Instead, the ‘Multi-End Europe’ model seems to be assessed very negatively in Germany because it is seen as a risk to the unity of the EU, while in France it is seen as a way to move integration forward among a core group of countries (Michel 2020; Nagel 2020). In other words, while France is focused on deepening integration, Germany is focused on keeping everyone on board. A possible explanation for Germany’s preference for an inclusive Europe lies in its export-oriented economic model, which relies on a large and open European market for both, cheap economic inputs and demand for German products (Schimmelfennig 2021). However, section “Integration preference: identity matters in the old member states” suggest that Germany’s highly Europeanized citizenry provides a plausible alternative explanation.

**Fig. 3:**
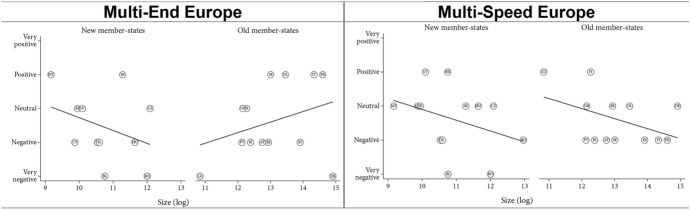
Dependence—old and new member states

Comparing old and new member states on the ‘Multi-End Europe’ model, the findings highlight a positive effect of size in the former (especially when excluding Germany), but a negative effect in the latter (which disappears when excluding Romania and Bulgaria). Regarding the ‘Multi-Speed Europe’ model, we found a negative effect of size for both groups (Fig. 3). Overall, these findings suggest a divergence of interests between large older member states, which tend to prefer permanent differentiation, and all smaller member states, which tend to prefer temporary differentiation. In other words, smaller member states seem to worry more about a loss of influence. This chimes with the prior insight that all types of opt-outs, even voluntary opt-outs, raise concerns about a potential loss of influence in Europe. Dependence matters.

#### Capacity: capacity matters in the new member states

**Fig. 4:**
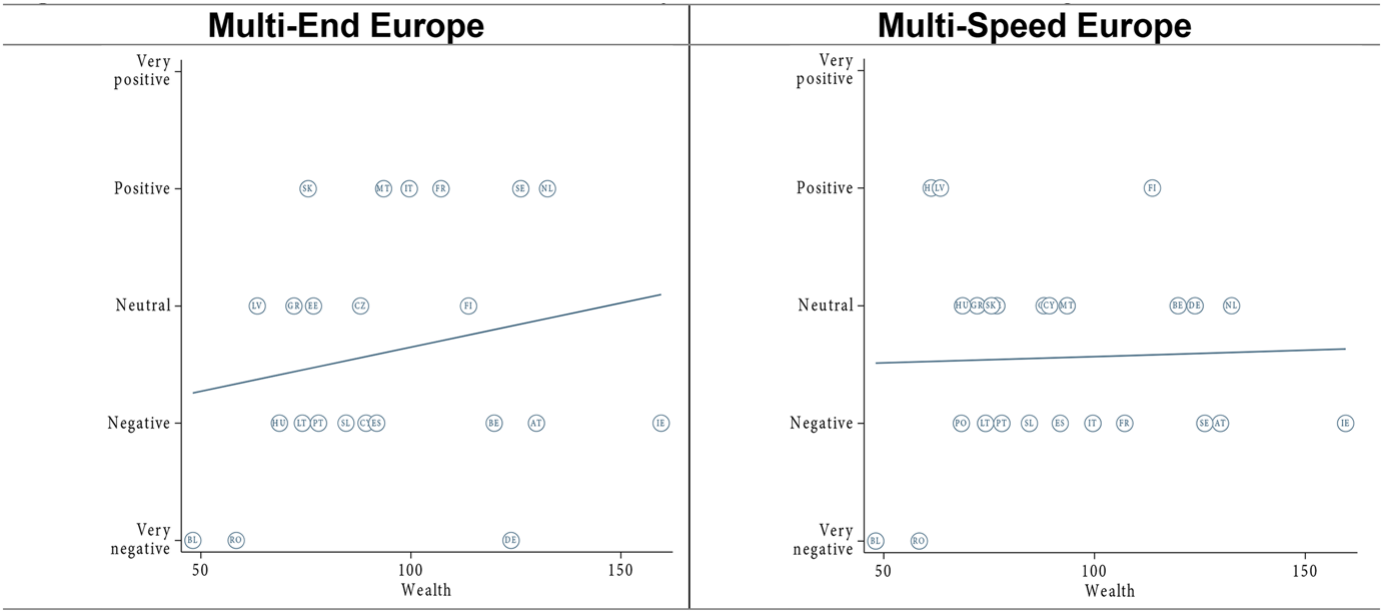
Effect of member state capacity (wealth, excl. Luxembourg)

In terms of capacity, we expected that both models would be viewed more negatively in poorer member states as compared to richer member states (H4a). We found very feeble evidence corroborating this expectation if we exclude Luxembourg from the analysis—by far the richest member state (Fig. 4, see also Appendix 6a). Like in Germany and Spain (Leon 2021; Nagel 2020), the ‘Multi-End Europe’ model is seen as a risk to the unity of the EU in Luxembourg (Michel 2021). At the same time, the ‘Multi-Speed Europe’ model is seen as a way to move integration forward, allowing Luxembourg to ‘act as an example of European integration and incentivise other Member States to follow its path’ (Michel 2021: summary). As such, the country’s small size and its highly Europeanized identity (see section “Integration preference: identity matters in the old member states”) appear to outweigh the effect of wealth. Finally, excluding Luxembourg, we did not find consistent evidence that poorer member states view the ‘Multi-End Europe’ model more negatively than the ‘Multi-Speed Europe’ model (H4b, Appendix 6b).

**Fig. 5:**
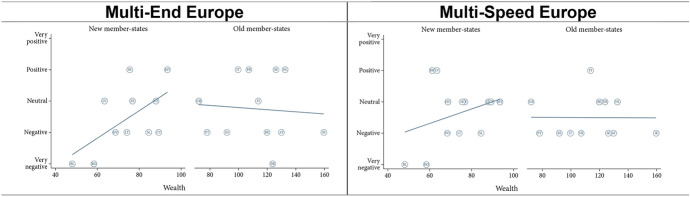
Capacity—old and new member states

However, we found interesting differences between old and new member states. In old member states, size appears to have no effect in orientations towards ‘Multi-Speed Europe’ model and only a very small effect for the ‘Multi-End Europe’ model (Fig. 5, see also Appendix 6c). By contrast, for the new member states, attitudes are generally more favourable in both models for wealthier countries. However, if we exclude Romania and Bulgaria, the correlation is much weaker. These two countries are indeed poorer than other new member states, but they are also subject to long-term involuntary opt-outs. Overall, capacity seems to matter more in the new member states than in the old member states.

#### Integration preference: identity matters in the old member states

**Fig. 6:**
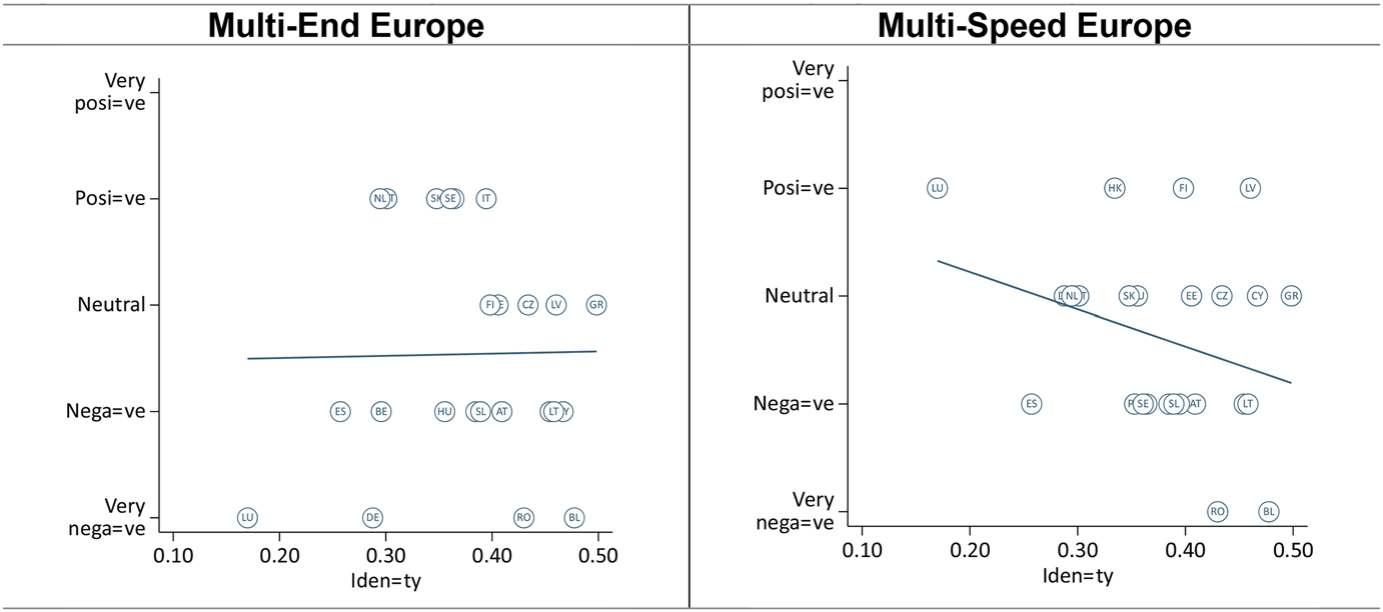
Effect of identity (share of citizens identifying as exclusively national)

Finally, we expected that the ‘Multi-End Europe’ model would be seen more positively in member states with more nation-identifying citizen than in less national member states (H5a). Vice versa, we expected that the latter would view the ‘Multi-Speed Europe’ model more positively (H5b). We found some evidence for the second, but not for the first hypothesis (Fig. 6).

**Fig. 7:**
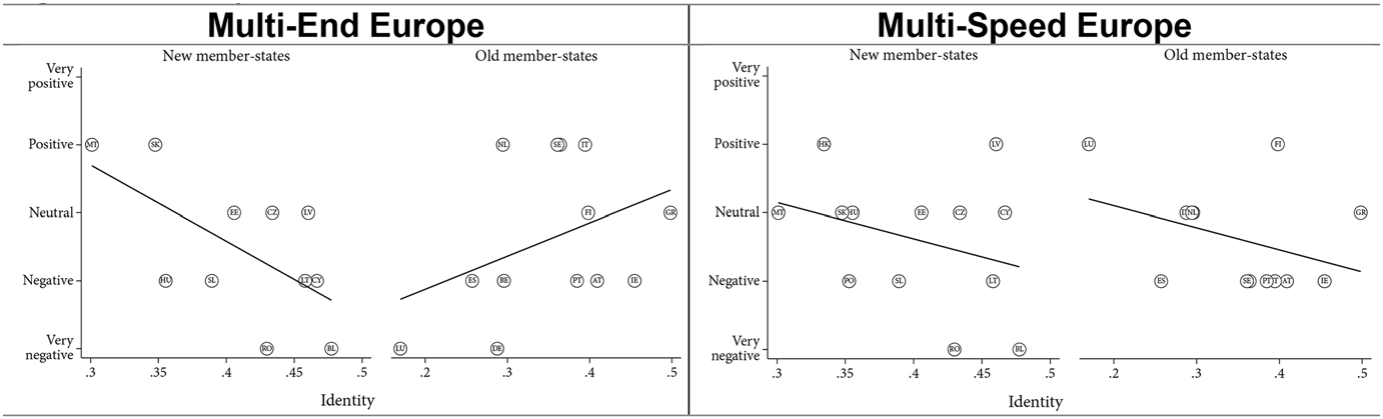
Identity—old and new member states

Comparing old and new member states again, member states with stronger national views assess the ‘Multi-Speed Europe’ model more negatively in both groups. However, with regard to the ‘Multi-End Europe’ model, the two groups strongly diverge (Fig. 7). More national member states view ‘Multi-End Europe’ more positively whereas older member states view it more negatively. One explanation may be that the most national *new* member states are also the poorest and/or experienced pro-longed involuntary exclusion from key EU policies. This suggests that the post-functionalist mechanisms—whereby public attitudes influence government positions about European integration—are indeed at work in the *older* member states but not in the newer member states.

## Discussion and conclusion

This paper investigated how temporary and permanent polity differentiation are assessed in the member states of the EU. It did so by analysing novel data collected from governmental documents and parliamentary minutes. We stress once more that we need to be cautious in interpreting the results of our analysis because, given the low salience of DI, national decision-makers may not necessarily have clear and stable positions on the issue.

And indeed, we did not find support for the normatively and functionally plausible hypothesis (H1) that the ‘Multi-Speed Europe’ model of temporary differentiation would be assessed more positively than the ‘Multi-End Europe’ model of permanent differentiation. Nevertheless, we found that two models are assessed differently in most member states, giving substance to the notion that politicians do distinguish between different types of polity differentiation.

We then investigated a variety of factors which could explain differences in the assessment of polity differentiation between the member states. We first looked at different forms of opt-outs and found no evidence that voluntary opt-outs are associated with more positive assessments of the ‘Multi-End Europe’ model (H2a). The reasons might be that the long-term effects of policy opt-outs are seen as negatively impacting influence in the EU polity, as our qualitative data suggest. By contrast, we did find evidence that long-term, ongoing involuntary opt-outs from key EU policies are associated with negative assessments of both models of polity differentiation (H2b). The evidence was weaker for the positive effect of ceased involuntary opt-outs (H2c), as politicians in several member states of this group often expressed the view that despite having overcome involuntary opt-outs, their country did still not belong to the ‘core’ of the EU.

Next, we looked at how member state dependence, capacity, and identity impact assessments of polity differentiation. The key finding here is that these factors seem to play out differently in old and new member states. Concerning dependence, the ‘Multi-End Europe’ model is seen more positively in larger old member states than small older member states (H3a). However, we do not find this pattern for the newer member states. Concerning the ‘Multi-Speed Model’, we find that all (old and new) small member states assess the model more positively than all (old or new) large member states. Overall, small member states prefer the ‘Multi-Speed Europe’ model, while large member states prefer the ‘Multi-end Europe’ model (H3b).

Concerning capacity, we found evidence that wealthier new member states view the ‘Multi-End Europe’ and the ‘Multi-Speed Europe’ model more positively than poorer new member states (H4a). For old member states, wealth seems to play no role in attitudes towards DI. This might be the case because the wealthier new member states have, on average, made more progress towards the European core than the poorer new member states. In other words, for these countries, the experience of DI has been that of a facilitated entry into the Union. Overall, we found little evidence that poorer member states prefer the ‘Multi-Speed Europe’ model over the ‘Multi-End Europe’ model (H4b).

Concerning identity, we found evidence that member states with a lower share of citizens identifying as exclusively national assess the ‘Multi-Speed Europe’ more positively than member states with a higher share of citizens identifying in this way (H5b). At the same time, we found little evidence that more national member states prefer the ‘Multi-End Europe’ model (H5a). However, we did find a rather strong positive correlation when we only focused on the old member states. By contrast, for the new member states, we found a negative correlation. This suggests that the post-functionalist mechanism matters in the old, but not (yet) in the new member states.

What explains these differences between old and new member states? As a group, newer member states are arguably more dependent on the EU—for economic development and political transformation—than older member states. As a corollary, the difference in dependence between large new member states and small new member states may be less crucial compared to the differences in dependence in the group of old member states. At the same time, capacity may matter more in new member states as capacity improvements are associated with accession to the ‘core’ of the EU. Long-term involuntary opt-outs only affects positions in (some of) the new member states. Moreover, the post-functionalist mechanism may be active only in the old member states because their publics there are more aware of European affairs.

Our research has several limitations which future research could address. First, our quantitative data are highly aggregated and did not allow us to establish causality statistically. We addressed this gap by enriching the quantitative findings with qualitative insights. However, for a more fine-grained analysis, it would be extremely useful to gather time series data and to code for additional factors such as governmental ideology or participation. Second, we have shown evidence that differentiated integration is not a high salience issue in most member states for most of the time. However, we have not investigated the relationship between salience and (the blur of) member state positions on polity differentiation. Third, as we have shown in the paper, time is merely one dimension of polity differentiation. Future research could investigate other dimensions, such as how member states assess the participation of non-members in EU policies or how cooperation among member states outside the treaties is assessed. And finally, our paper does not sufficiently problematize the link between popular and elite attitudes about DI. Considering that recent research on this issue comes to varying conclusions, showing alternatively that demographic characteristics matter for how citizens view DI (Leuffen et al. 2021) and that citizen’s views are shaped by elite perceptions (Telle et al. 2022), this promises to be a fruitful field for future research.

## Appendices

## Appendix 1: Keywords for polity differentiation

**Table:** 

Multi-speed EU	Multi-end EU
• two-speed europe/eu • multi-speed europe/eu • coalition of the willing	• variable geometry • core europe/european core • two-tier europe • concentric circles + eu • a la carte + eu

## Appendix 2: Overview of document categories analysed

**Table:** 

	Category of document	Time period	Instructions
1	Government programmes	2004*–2020	Find repository or use search engine to retrieve government programmes/coalition agreements
2	First speeches (and parliamentary debate)	2004*–2020	Retrieve the first speech after election of each PM/President in parliament and the subsequent debates
3	Council of EU Presidency speeches (and parliamentary debate) a. in National Parliament b. in European Parliament	2004*–2020	Retrieve the first speech during the EU Council Presidency of each PM/President in the national and the European parliament (and the immediately following parliamentary debate)
4	Future of Europe speeches (and parliamentary debate) a. in European Parliament b. for citizen consultation	2017–2020	Retrieve the PM/HS speech in the European Parliament on the “Future of Europe” Retrieve a PM speech on the citizen consultation on the “Future of Europe”
5	Prime Minister European Council Statements	2004*-2020	Search repository and retrieve all pre- and post-Council statements of the PM
6	Parliamentary (committee) debates	2004–2020	Search repository of parliamentary debates, using keywords
7	If category 6 is not feasible, collect miscellaneous documents referring to DI	as needed	Use keywords in search engine to retrieve documents which refer to DI: press releases, government reports, social media posts, media interviews, PM speeches (i.e. Macron 2017), etc

## Appendix 3: Case selection for opt-out experience

**Table:** 

*Opt-out type*	*Opt-out status*	*Schengen Area* *Opt-out countries*	*Eurozone* *Opt-out countries*	*Expectation*
*Voluntary* *(exemptive)*	*Ongoing*	**Denmark** **UK** Ireland	**Denmark** **UK** *Sweden (*de facto*)* *Czech Republic (*de facto*)* *Hungary (*de facto*)* *Poland (*de facto*)*	Positive assessment of ‘Multi-End Europe’
*Involuntary* *(discriminatory)*	*Ongoing (and potentially permanent)*	**Romania (2007-)** **Bulgaria (2007-)** **Croatia (2013-)** Cyprus (2004-)	**Romania (2007-)** **Bulgaria (2007-)** **Croatia (2013-)**	Negative assessment of ‘Multi-Speed Europe’ and Multi-End Europe’
	*Ceased*	Italy (1995–1997) **Greece (1995–2000)** Austria (1995–1997) Finland (1996–2001) *Sweden (1996–2001)* *Czech Republic (2003–2007)* *Hungary (2003–2007)* *Poland (2003–2007)* **Malta (2003–2007)** **Slovakia (2003–2007)** **Slovenia (2003–2007)** **Latvia (2003–2007)** **Lithuania (2003–2007)** **Estonia (2003–2007)**	**Malta (2004–2008)** **Slovakia (2004–2009)** **Slovenia (2004–2007)** **Latvia (2004–2014)** **Lithuania (2004–2015)** **Estonia (2004–2011)** **Greece (1999–2001)** Cyprus (2004–2008)	Positive assessment of ‘Multi-Speed Europe’

Bold = most likely cases; italics = complementary experience; underlined = neutralizing experience for ‘Multi-Speed Europe’. The involuntary exclusion of Cyprus from the Schengen Area is linked to the de facto partition of the island following the 1974 Turkish invasion.

## Appendix 4: Salience of DI by country


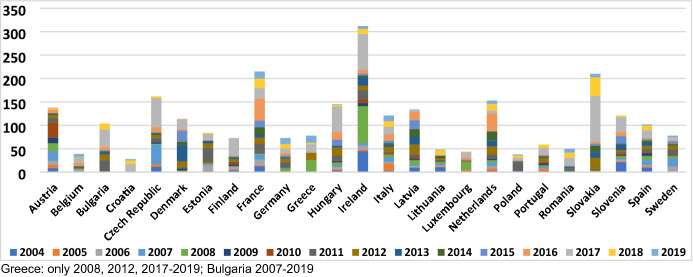


## Appendix 5: Small member states prefer ‘Multi-Speed’, large member states prefer ‘Multi-End’


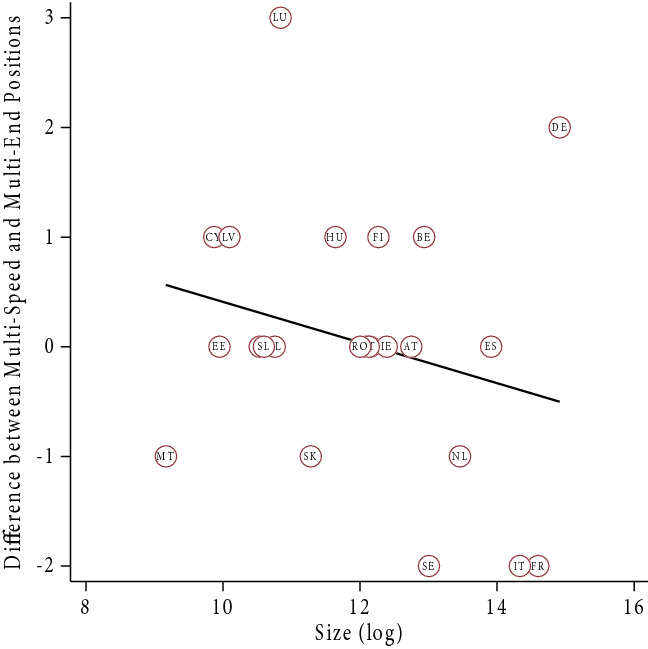


## Appendix 6a: Effect of capacity on positions about polity differentiation (incl. Luxembourg)


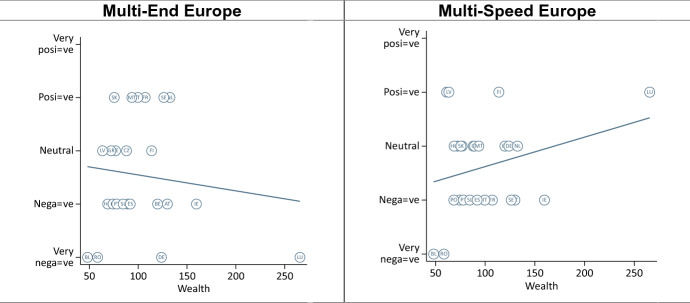


## Appendix 6b: Do poorer member states prefer ‘Multi-Speed Europe’?


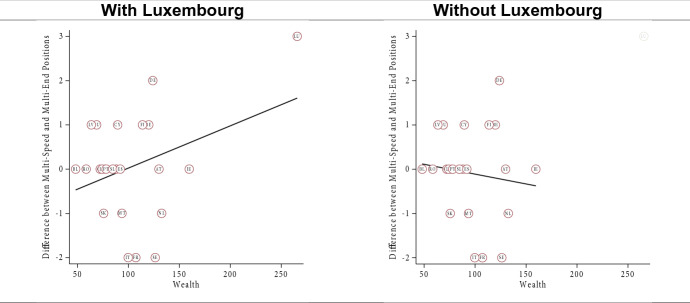


## Appendix 6c: Capacity Old vs. New Member States (incl. Luxembourg)


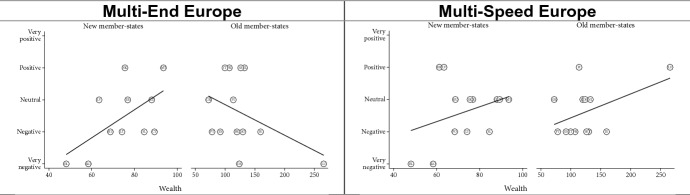


## Footnotes

1 In fact, according to Leuffen et al. (2013; Schimmelfennig et al. 2015), an ‘Europe a la carte’ is not really a system of differentiated integration but a system of overlapping and competing jurisdictions (FOCJ). This is the case because in this model, membership in policy alliances strongly varies from policy to policy, leaving the EU without a core.

2 The activation of the post-functionalist mechanism depends on the politicization of European Affairs in the domestic political sphere. Schimmelfennig, Leuffen, and Rittberger (2015) suggest that high politicization explains horizontal differentiation in cases of high interdependence in a specific policy field. Research has shown that the politicization of the EU is generally low but varies from country to country and over time (Hutter et al. 2016; Kriesi 2016). Hence, if we do not find the expected relationships to hold, this might suggest low or uneven levels of politicization of the EU in the member states.

3 The attribution of specific key words to the two models of differentiated integration is based on the authors’ reading of the academic literature and the empirical data. As such, it cannot be excluded that national decision-makers always attribute a different meaning to the key words. Instances in which the usage of key words by politicians suggests that this might be the case are discussed in “Dependence, capacity, and identity” section.

4 Eurostat_GDP.

5 Eurostat_GDP per capita.

6 Eurobarometer_identity.

7 No data for Denmark.

8 No data for Croatia, Denmark, and Poland.

9 On a scale from 1 (very negative) to 5 (very positive), the ‘Multi-Speed Europe’ model scores 2,62 while the ‘Multi-End Europe’ model scores 2.54 as an average of all available member state scores. Interestingly, old and new member states (accession before/after 2004) assess the ‘Multi-Speed Europe’ model identically (2.62). The ‘Multi-End Europe’ model is seen slightly more negatively in the group of new member states (OMS: 2.62 / NMS: 2.45).

10 The references in this section refer to the country reports which were referred to in Sect. 2. To distinguish between direct quotes of national decision-makers and interpretations by the authors of the reports, we use different reference schemes: For direct quotes (Name of speaker, place, date; cited in XXXX 2020), for interpretations by the authors of the country reports (XXXX 2020).
